# Precursor Amino Acids Inhibit Polymyxin E Biosynthesis in *Paenibacillus polymyxa*, Probably by Affecting the Expression of Polymyxin E Biosynthesis-Associated Genes

**DOI:** 10.1155/2015/690830

**Published:** 2015-05-21

**Authors:** Zhiliang Yu, Chenglin Guo, Juanping Qiu

**Affiliations:** College of Biological and Environmental Engineering, Zhejiang University of Technology, Hangzhou 310014, China

## Abstract

Polymyxin E belongs to cationic polypeptide antibiotic bearing four types of direct precursor amino acids including L-2,4-diaminobutyric acid (L-Dab), L-Leu, D-Leu, and L-Thr. The objective of this study is to evaluate the effect of addition of precursor amino acids during fermentation on polymyxin E biosynthesis in *Paenibacillus polymyxa*. The results showed that, after 35 h fermentation, addition of direct precursor amino acids to certain concentration significantly inhibited polymyxin E production and affected the expression of genes involved in its biosynthesis. L-Dab repressed the expression of polymyxin synthetase genes *pmxA* and *pmxE*, as well as 2,4-diaminobutyrate aminotransferase gene *ectB*; both L-Leu and D-Leu repressed the *pmxA* expression. In addition, L-Thr affected the expression of not only *pmxA*, but also regulatory genes *spo0A* and *abrB*. As L-Dab precursor, L-Asp repressed the expression of *ectB*, *pmxA*, and *pmxE*. Moreover, it affected the expression of *spo0A* and *abrB*. In contrast, L-Phe, a nonprecursor amino acid, had no obvious effect on polymyxin E biosynthesis and those biosynthesis-related genes expression. Taken together, our data demonstrated that addition of precursor amino acids during fermentation will inhibit polymyxin E production probably by affecting the expression of its biosynthesis-related genes.

## 1. Introduction

Polymyxin E is biosynthesized in* Paenibacillus polymyxa* [1]. It has been used as an important therapy for infection caused by Gram-negative pathogens since 1959. However, its clinical use was soon limited due to its adverse nephrotoxicity and neurotoxicity [2]. Recently, multidrug resistance in pathogens to almost all currently available antibiotics is becoming a big threat for human health, leaving very limited choices for clinical therapy [3–5]. In many cases, polymyxin E is considered as one of the last-line available options for treatment of multidrug-resistant bacteria [6, 7].

The basic structure of polymyxin E is a cyclic heptapeptide with a tripeptide side chain acylated by a fatty acid at the amino terminus [8, 9]. It is biosynthesized by a multienzyme nonribosomal peptide synthetase system (NRPS) [1, 10–12]. A gene cluster for its biosynthesis has been determined to have five open reading frames,* pmxA*,* pmxB*,* pmxE*,* pmxC*, and* pmxD*, encoding three polymyxin synthetases PmxA, PmxB, and PmxE, and two membrane transporters PmxC and PmxD, respectively [13–16]. PmxA comprises four modules whose amino acid substrates are D-Leu, L-Leu, L-2,4-diaminobutyric acid (L-Dab), and another L-Dab, while PmxB composes only one module with L-Thr as its amino acid substrate. PmxB is also responsible for synthesis termination. PmxE is another synthetase with five modules whose amino acid substrates are L-Dab, L-Thr, L-Dab, L-Dab, and L-Dab. In total, polymyxin E consists of ten orderly assembled amino acid residues or precursor amino acids [17]. Among them, six are L-Dabs which are biosynthesized by 2,4-diaminobutyrate aminotransferase (EctB) encoded by* ectB* [18]. It has been reported that addition of L-Dab to medium at the beginning of fermentation can stimulate polymyxin E production [19]. Moreover, addition of L-aspartic acid (L-Asp), Dab precursor, to medium in the presence of PO_4_^3−^ can also stimulate* P. polymyxa* to produce polymyxin E. In contrast, addition of L-Ile and L-Val gave suppression and the amino acids L-Thr and L-Leu gave no change in polymyxin production [19]. To date, there is no report on the molecular mechanism in affecting polymyxin E production upon addition of precursor amino acids during fermentation.

**Figure 1:**
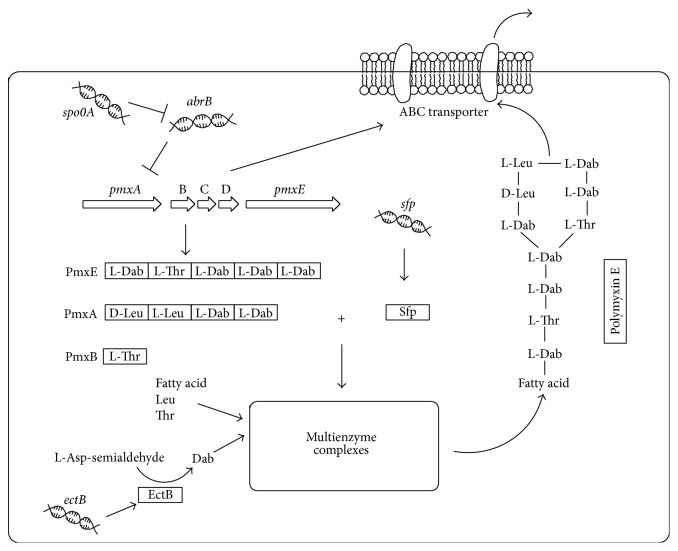
Relationships among the polymyxin E biosynthesis-related genes [13–17, 27]. Polymyxin E is biosynthesized by a multienzyme system named NRPS, which is comprised of three synthetases PmxA, PmxB, and PmxE encoded by* pmxA*,* pmxB*, and* pmxE*, respectively. PmxA comprises four modules whose amino acid substrates are D-Leu, L-Leu, L-Dab, and L-Dab. PmxB composes only one module with L-Thr as its amino acid substrate. PmxE has five modules whose amino acid substrates are L-Dab, L-Thr, L-Dab, L-Dab, and L-Dab. Based on the polymyxin E structure, the order of modules for amino acid assembly during polymyxin E synthesis is PmxE-PmxA-PmxB, which is consistent with the order of ten amino acid groups on polymyxin E molecule. The multienzyme system is probed and activated by phosphopantetheinyl transferase (Sfp) encoded by* sfp*. The* pmxA* expression is negatively regulated by a DNA-binding protein, AbrB encoded by* abrB*. The* abrB* expression is negatively regulated by another DNA-binding protein, Spo0A encoded by* spo0A*. Polymyxin E is secreted by ABC transporters PmxC and PmxD encoded by* pmxC* and* pmxD*, respectively. Dab is synthesized from L-asp-semialdehyde (L-Asp) by EctB which is encoded by* ectB*. Fatty acid: 6-methyloctanoic acid or isooctanoic acid; Thr: threonine; Phe: phenylalanine; Leu: leucine; and Dab: *α*,*γ*-diaminobutyric acid.

It has been reported that polymyxin E biosynthesis is negatively regulated by AbrB, a DNA-binding protein, by directly binding to the upstream region of* pmxA *[18]. AbrB itself is an unstable protein and its gene expression is negatively regulated by Spo0A, another DNA-binding protein encoded by* spo0A* [20–23]. Besides these two regulation proteins, phosphopantetheinyl transferase (Sfp) encoded by* sfp* was found to be important in priming and activating NRPS [24–26]. All these genes together with the ones in gene cluster are believed to be important for polymyxin E biosynthesis and their relationships are outlined in Figure 1 [13–17, 27].

In this study, we aim to investigate the effect of precursor amino acids added during fermentation on polymyxin E production in* P. polymyxa*. We found that addition of precursor amino acids will suppress polymyxin E production probably by affecting the expression of polymyxin E biosynthesis-related genes. We believe that this study would be useful for understanding the regulation mechanism underlying polymyxin E biosynthesis during fermentation.

## 2. Materials and Methods

### 2.1. Strain and Culture Conditions

As the producer of polymyxin E,* P. polymyxa* used in this study was supplied by Zhejiang Qianjiang Biochemical Co., Ltd., China, and frozen at −80°C in our lab at Zhejiang University of Technology, China. Unless otherwise specified,* P. polymyxa* was firstly cultivated on agar plate (10 g/L of beef extract, 15 g/L of peptone, 10 g/L of glucose, 2 g/L of yeast extract, 3 g/L of NaCl, 0.1 g/L of FeSO_4_·7H_2_O, and 20 g/L of agar) at 30°C for 2 d. Then, a ring of* P. polymyxa* was transferred to 50 mL of seed medium (30 g/L of soybean meal, 5 g/L of soybean oil, 0.1 g/L of FeSO_4_·7H_2_O, 15 g/L (NH_4_)_2_SO_4_, 0.77 g/L of KH_2_PO_4_, 0.7 g/L of CaCO_3_, and 10 g/L of glucose) in 250 mL flask for incubation at 30°C for 24 h with shaking at 200 rpm. Next, 5 mL of cell culture was transferred to 50 mL of fermentation medium (23.9 g/L of soybean meal, 21.1 g/L of soybean meal powder, 10 g/L of soybean oil, 0.1 g/L of FeSO_4_·7H_2_O, 25 g/L (NH_4_)_2_SO_4_, 0.77 g/L of KH_2_PO_4_, 1 g/L of CaCO_3_, and 25 g/L of glucose) in 250 mL flask at 30°C with shaking at 200 rpm for fermentation. After fermentation with various lengths, the concentration of polymyxin E was measured by HPLC and the gene expression was detected by quantitative real-time PCR (qRT-PCR).

### 2.2. Treatment of Precursor Amino Acids

To evaluate the effect of precursor amino acids on polymyxin E production and its biosynthesis-related genes expression, after 35 h fermentation, different precursor amino acids with various concentrations were added to culture for stimulation. After a further 8 h fermentation, the concentration of polymyxin E was measured by HPLC and the gene expression was analyzed by qRT-PCR.

### 2.3. HPLC Analysis of Polymyxin E

One milliliter of culture was centrifuged at 10,000 g for 10 min and the supernatant was collected after filtration with 0.45 *μ*m microporous membrane. Analysis was performed using an HPLC system (SHIMADZU, Japan). 20 *μ*L of supernatant sample was injected into a reverse-phase column, YMC Pack ODS-A (150 × 4.6 mm I.D., 5 *μ*m), eluted at 33°C, and analyzed in a mixed solvent of acetonitrile (22%) and water containing 0.223% Na_2_SO_4_ (78%), at a constant flow of 1 mL/min. Peak of polymyxin E was determined at wavelength of 240 nm. Polymyxin E concentration produced by* P. polymyxa* was calculated based on the extracted correlation equation between the concentration of standard polymyxin E and the corresponding peak area in HPLC. One unit is equal to 0.0418 *μ*g of polymyxin E.

### 2.4. PCR Amplification and Sequence Collection of Genes Associated with Polymyxin E Biosynthesis

The partial sequences of* pmxABCDE*,* spo0A*,* abrB*,* ectB*, and* sfp* were amplified by PCR. The primers (Table 1) used for PCR were designed based on the complete genome sequence of* Paenibacillus polymyxa* E681 (GenBank number CP000154) [13]. The bacterial genomic DNA was extracted using a bacterial genomic DNA extraction kit (GE, USA). The gene fragments were amplified in 50 *μ*L containing 37 *μ*L of ddH_2_O, 5 *μ*L of 10x Easy* Taq* buffer, 4 *μ*L of 2.5 mM dNTPs, 100 nM forward primer, 100 nM reverse primer, 1 ng genomic DNA, and 1 U* Taq* DNA polymerase (TaKaRa, Dalian, China) with denaturation at 94°C for 5 min followed by 30 cycles of 1 min at 94°C, 50 s at 55°C, 90 s at 72°C, and a final 10 min extension at 72°C. At the end of reaction, PCR product was cooled to 4°C for further use. After size confirmation on 1.0% agarose gel, the desired amplicons were purified using a gel extraction kit (Qiagen, CA, USA) for TA cloning with pMD19-T simple vector (TaKaRa, Dalian, China). After sequencing by Sangon Biotech (Shanghai, China), the gene sequences were collected and compared with the reference genes in GenBank for confirmation.

### 2.5. Analysis of Gene Expression Using qRT-PCR

To measure gene expression, qRT-PCR [28] was used to amplify cDNA products reversely transcribed from mRNA. In brief, the bacterial cell was harvested through centrifugation at 5,000 rpm for 5 min and the total RNA was extracted using an RNAiso Plus kit (Sangon, Shanghai, China). RNA integrity was determined based on the OD_260 nm_/OD_280 nm_ ratio (>1.95), and 500 ng of DNA-free RNA with high-quality was reversely transcribed to cDNA in a 10 *μ*L volume using PrimeScript RT Master Mix (Perfect Real Time) kit. After appropriate dilution, the cDNA was used for amplification of target gene fragment with primer sets (Table 2) by using the SYBR green* Premix Ex Taq* (Tli RNaseH Plus) kit. PCR was run on CFX Connect Real-Time System (Bio-Rad, Hercules, CA) with an amplification protocol consisting of an initial denaturation at 95°C for 10 min, followed by 40 cycles of denaturation at 95°C for 15 s and annealing/elongation at 60°C for 30 s. Immediately after the final PCR cycle, a melting-curve analysis was made to determine the reaction specificity based on the observed melting temperature from product. Unless otherwise specified, all the kits above were purchased from TaKaRa Bio. Inc. (Dalian, China).

The cycle threshold (*C*_*T*_) for each PCR was determined using StatView software which automatically sets the threshold signal at the log phase of amplification curve. Several dilutions of each cDNA sample were assayed for gene of interest in order to obtain a linear regression between the *C*_*T*_ values (ranging from 20 to 35 cycles) and the log of cDNA. The amplification efficiency of gene was retrieved from the slope of that linear regression according to the formula *E* = 10^(−1/slope)^. The 116 bp of housekeeping 16S rRNA gene fragment was amplified by using primer set of 16SF (5′-GAGAAGAAAGCCCCGGCTAA-3′) and 16SR (5′-ACCAGACTTAAAGAGCCGCC-3′) and treated as the internal control to verify that there were equal amounts of target cDNA in all samples. The relative expression of the target gene compared to that of the reference 16S rRNA gene was calculated by comparative *C*_*T*_ method [29].

### 2.6. Data Analysis

All data were presented as mean ± standard error and tested for statistical significance based on analysis of variance (ANOVA) followed by Dunnett's post hoc test using StatView 5.0 program. When the probability (*P*) was less than 0.05 and 0.01, the values were considered significantly and very significantly different, respectively.

## 3. Results

### 3.1. Accumulation of Polymyxin E during Fermentation

**Figure 2:**
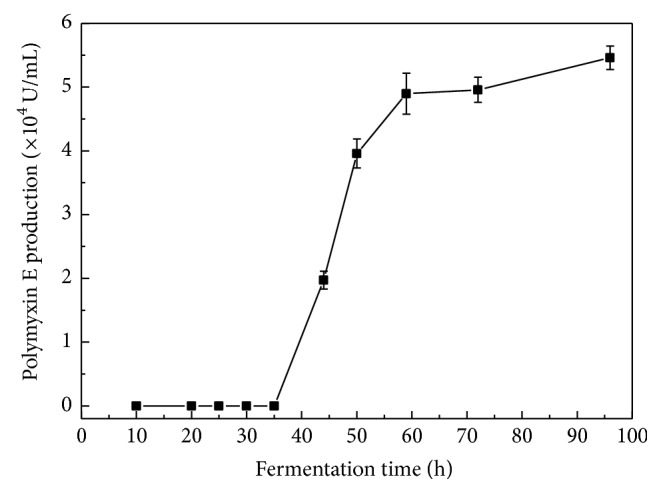
Polymyxin E accumulation in* P. polymyxa* during fermentation.

Polymyxin E produced by* P. polymyxa* was measured over a 96 h fermentation period using HPLC. As shown in Figure 2, no polymyxin E was detected within 35 h. Then, its production rapidly increased from 35 h to 59 h. Next, polymyxin E production kept almost constant in the remaining time frame. Thus, under these fermentation conditions, polymyxin E is mainly produced by* P. polymyxa* over a period from 35 h to 59 h. Therefore, in the following experiments, a fermentation period from 35 h to 72 h was selected for monitoring polymyxin E production and the expression of genes involved in polymyxin E biosynthesis.

### 3.2. Expression of Genes Involved in Polymyxin E Biosynthesis

**Figure 3:**
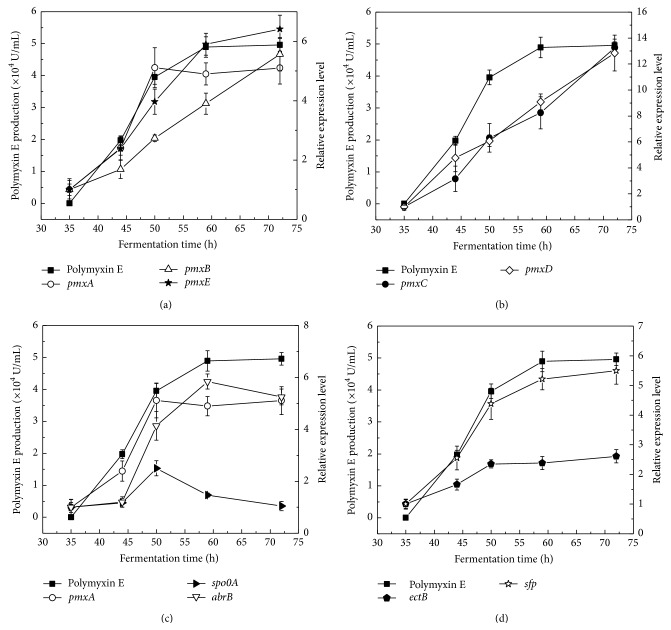
Polymyxin E accumulation and relative expression of genes including* pmxA*,* pmxB*, and* pmxE* (a),* pmxC* and* pmxD* (b),* pmxA*,* spo0A*, and* abrB* (c), and* ectB* and* sfp* (d).

Figure 3(a) showed that the* pmxA* expression increased rapidly over a period from 35 h to 50 h, in line with the increase of polymyxin E production. Then,* pmxA* expression remained almost unchangeable till 72 h, while polymyxin E production had a further increase from 50 h to 59 h and then stayed constant. Over an entire 72 h fermentation period, the expression of another two polymyxin E synthetase genes,* pmxB* and* pmxE*, as well as* pmxC* and* pmxD* genes kept increasing (Figures 3(a) and 3(b)). Similarly, the expression of both* ectB* and* sfp* kept increasing (Figure 3(d)). The expression of* abrB*, a negative regulator gene for* pmxA*, increased very slightly from 35 h to 45 h, but rapidly from 45 h to 50 h. After a further increase till 59 h, its expression turned to decrease (Figure 3(c)). The expression of* spo0A*, another important regulator gene for* abrB*, also increased very slightly from 35 h to 45 h and rapidly from 35 h to 50 h, but its expression turned to decrease at 50 h (Figure 3(c)). Therefore, there exists a delay in the change pattern between* abrB* expression and* spo0A* expression. Most probably, Spo0A needs phosphorylation before negative regulation on* abrB* expression [21, 22]. All these results indicated that polymyxin E biosynthesis is probably limited due to the repression of* pmxA* expression, which has probably resulted from AbrB accumulation, consistent with the reports [20–23].

### 3.3. Repression of Polymyxin E Production by Precursor Amino Acids

**Figure 4:**
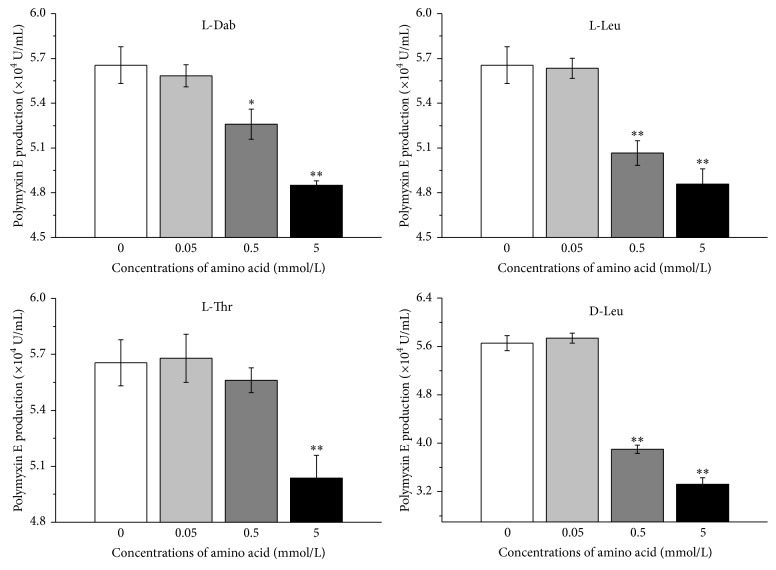
Effect of precursor amino acids on polymyxin E production. “*∗*” and “*∗∗*” represent a statistically significant (*P* < 0.05) and very significant (*P* < 0.01) difference, respectively, when compared to the control without addition of precursor amino acid at 35 h.

Polymyxin E requires precursor amino acids including L-Dab, L-Leu, D-Leu, and L-Thr for its biosynthesis.  Figure 4 showed that, compared to control without addition of precursor amino acids, the treatments with precursor amino acids at low concentration (0.05 mmol/L) all had no significant effect on polymyxin E production. However, addition of precursor amino acids to higher concentration repressed the polymyxin E production. In general, the higher the concentration of precursor amino acids from 0.5 mmol/L to 5 mmol/L, the stronger the repression to polymyxin E production. L-Dab at 0.5 mmol/L significantly (*P* < 0.05) decreased polymyxin E production. Similarly, L-Leu and D-Leu at 0.5 mmol/L as well as L-Thr, L-Leu, and D-Leu at 5 mmol/L all very significantly (*P* < 0.01) suppressed polymyxin E production.

### 3.4. Regulation of Precursor Amino Acids on the Expression of Genes Involved in Polymyxin E Biosynthesis

**Figure 5:**
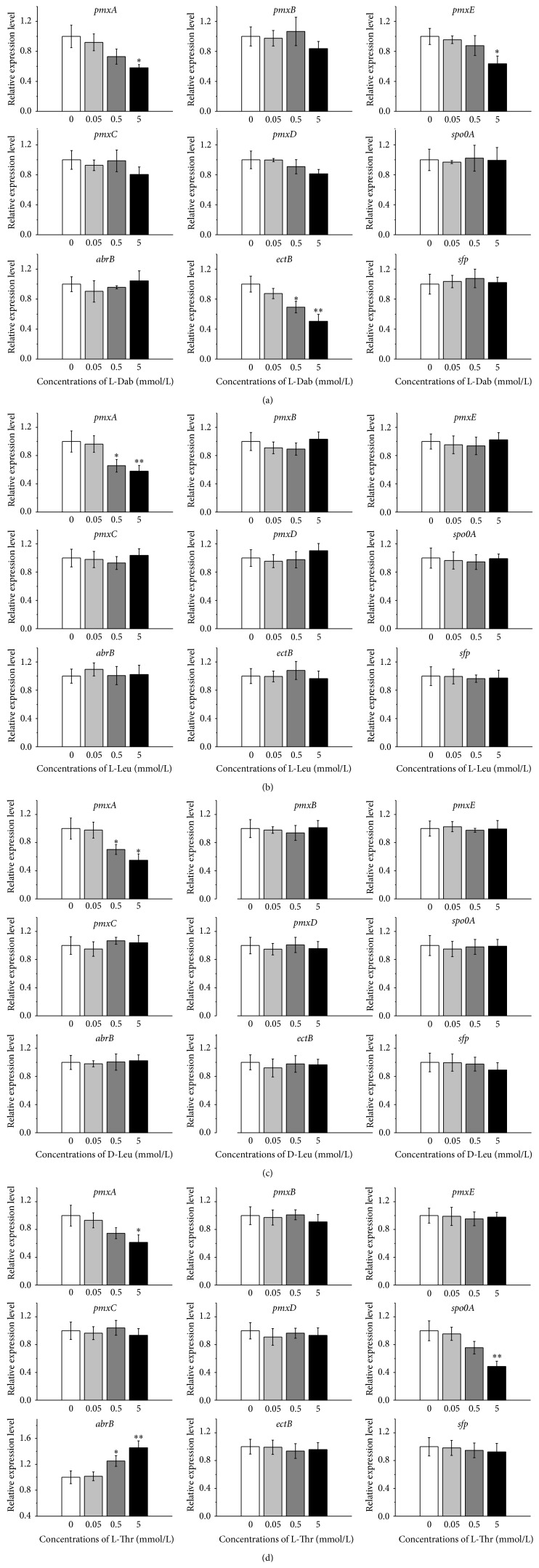
Effect of precursor amino acids on the expression of genes involved in polymyxin E synthesis. (a) L-Dab; (b) L-Leu; (c) D-Leu; and (d) L-Thr.

L-Dab is biosynthesized by EctB in cells [30]. As shown in Figure 5(a), L-Dab at 0.05 mmol/L had no significant impact on* ectB* expression, while the ones at both 0.5 mmol/L and 5 mmol/L significantly (*P* < 0.05) and very significantly (*P* < 0.01) downregulated the* ectB* expression, respectively. In addition, as substrate of both PmxA and PmxE, L-Dab at 5 mmol/L significantly reduced their coding genes expression. On the contrary, other genes' expression was almost unaffected by L-Dab.

Both L-Leu and D-Leu are substrate amino acids of PmxA for polymyxin E biosynthesis. It was shown that L-Leu at 0.5 mmol/L and 5 mmol/L significantly (*P* < 0.05) and very significantly (*P* < 0.01) repressed the* pmxA* expression, respectively. Similarly, D-Leu at 0.5 mmol/L and 5 mmol/L both significantly (*P* < 0.05) reduced the* pmxA* expression. However, they had no significant impact on other genes' expression (Figures 5(b) and 5(c)).

Interestingly, as substrate amino acid of both PmxB and PmxE, L-Thr had no significant effect on either* pmxB* or* pmxE* expression (Figure 5(d)) but surprisingly affected the expression of* abrB*,* spo0A*, and* pmxA*. L-Thr at 0.5 mmol/L caused significant (*P* < 0.05) increase in* abrB* expression. Moreover, L-Thr at 5 mmol/L significantly (*P* < 0.05) and very significantly (*P* < 0.01) downregulated the expression of* pmxA* and* spo0A*, respectively, and very significantly (*P* < 0.01) upregulated the* abrB* expression.

### 3.5. Effect of Indirect-Precursor Amino Acid on Polymyxin E Production and the Expression of Genes Involved in Polymyxin E Synthesis

**Figure 6:**
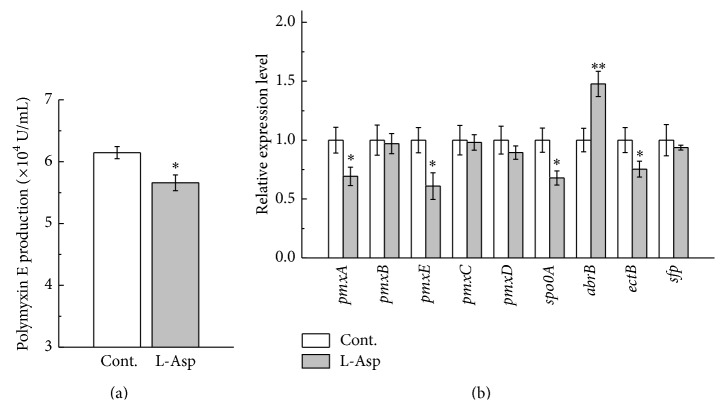
Effect of L-Asp on polymyxin E production (a) and the expression of genes involved in polymyxin E biosynthesis (b). Cont.: control group (fermentation without L-Asp addition).

L-Dab is biosynthesized from L-Asp in cells. Therefore, L-Asp was chosen as an indirect-precursor amino acid in polymyxin E biosynthesis. As shown in Figure 6(a), L-Asp at 5 mmol/L also significantly (*P* < 0.05) decreased the polymyxin E production. Data in Figure 6(b) further showed that L-Asp at 5 mmol/L significantly (*P* < 0.05) repressed the expression of* pmxA*,* pmxE*,* spo0A*, and* ectB* and very significantly (*P* < 0.01) stimulated the* abrB* expression.

### 3.6. Effect of Nonprecursor Amino Acid on Polymyxin E Production and the Expression of Genes Involved in Polymyxin E Biosynthesis

**Figure 7:**
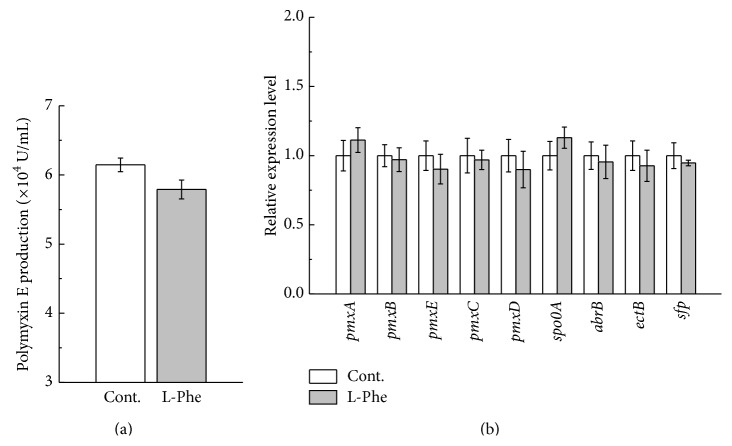
Effect of nonprecursor amino acid of L-Phe on polymyxin E production (a) and the expression of genes involved in polymyxin E biosynthesis (b). Cont.: control group (fermentation without L-Phe addition).

L-Phe was chosen as a representative of nonprecursor amino acids for polymyxin E biosynthesis. As shown in Figure 7, 5 mmol/L of L-Phe had no significant influence on both polymyxin E accumulation and the expression of polymyxin E biosynthesis-associated genes.

## 4. Discussion

It has been reported that AbrB repressed the expression of* tycA* encoding tyrocidine synthetase I, an enzyme for cyclic decapeptide tyrocidine biosynthesis in* Bacillus brevis* [23]. Another report showed that* abrB* deletion strongly increased lantibiotic subtilin production in* Bacillus subtilis* [31]. Recently, AbrB was found to directly bind to the upstream region of* pmxA* for negative regulation on polymyxin biosynthesis [20]. It was believed that negative regulation function of AbrB is probably due to its direct binding to promoter of the genes [20]. Spo0A also functions as a regulator of many genes through repression of* abrB* expression [21, 22]. In* Bacillus cereus*, cereulide production was regulated by Spo0A and AbrB through controlling the expression of its biosynthesis gene [30]. Our study showed that the expression of genes* pmxA*,* abrB*, and* spo0A* together with polymyxin E production in* P. polymyxa* all increased from 35 h to 50 h. The* spo0A* expression would result in Spo0A accumulation which probably suppressed the* abrB* expression at 59 h. Spo0A needs phosphorylation before negative regulation on* abrB* expression [21, 22], explaining the delay in the change pattern between the* abrB* and* spo0A* expression. Similarly, the* abrB* expression from 35 h to 50 h may cause AbrB accumulation to a certain concentration level which subsequently repressed the* pmxA* expression at 50 h probably by directly binding to upstream region of* pmxA* [18]. This led to suppression of polymyxin E biosynthesis at 59 h.

Polymyxin E is a secondary metabolite, which is supported by our result that it accumulated mainly over a period from 35 h to 59 h (Figure 2). Though addition of either Dab or its precursor L-Asp to medium at the beginning of fermentation has been shown to stimulate polymyxin E production [19], our results showed that, after 35 h fermentation, the addition of precursor amino acids to high concentration all repressed polymyxin E production (Figure 4). There are several possible explanations for this inconsistency. Most probably, the fermentation medium for addition of precursor amino acids, addition moment of precursor amino acids, the used producer strain, and/or concentration of precursor amino acids are different between the two cases. The mimic experiment by using the same condition [19] besides strain further showed that L-Dab addition to medium M [19] also caused a reduced polymyxin E production (data not shown). Therefore, this discrepancy most probably attributes to the difference of the used strain. As substrate amino acids of PmxA, L-Dab, L-Leu, and D-Leu significantly repressed the* pmxA* expression. Similarly, as substrate amino acid of PmxE, L-Dab had significant repression on* pmxE* expression. However, L-Thr, the substrate amino acid of both PmxB and PmxE, significantly affected the expression of regulation genes* spo0A* and* abrB*, instead of* pmxB* and* pmxE*. Most probably, L-Thr suppressed the* spo0A* expression and stimulated the* abrB* expression, eventually leading to the repression of* pmxA* expression. Accordingly, polymyxin E production was inhibited. Polymyxin E includes 6 L-Dabs synthesized by EctB [32, 33]. Our data indicated that the* ectB* expression decreased after addition of L-Dab to 5 mmol/L, suggesting that L-Dab at high concentration can downregulate the* ectB* expression. L-Dab is biosynthesized from L-Asp. It was found that L-Asp also affected the* ectB* expression. Interestingly, L-Asp repressed the expression of both* pmxA* and* pmxE*. Moreover, L-Asp increased and decreased the expression of* spo0A* and* abrB*, respectively. Most probably, L-Asp addition will enhance the L-Dab concentration in cells, accordingly affecting the L-Dab-associated genes expression. In contrast, all the precursor amino acids unaffected the expression of* pmxC* and* pmxD* as well as* sfp*.

To our best knowledge, it is the first time to investigate the effect of precursor amino acids on polymyxin E production and its biosynthesis-associated genes expression. This is helpful for understanding the regulation mechanism for polymyxin E biosynthesis during fermentation.

## Figures and Tables

**Table 1:** Sequences of primer pairs for PCR amplification of genes involved in polymyxin E biosynthesis.

Genes	Nucleotide sequences (5′-3′)		Product sizes (bp)
	Forward primers	Reverse primers	
*pmxA *	CCGCCCATTATGACAACCGT	GCTGGCGAATTGAACGATCC	1238
*pmxB *	ATGAAATCTTTGTTTGAAAA	CCAGGACGTACACCCTCAAC	1878
*pmxC *	AAAGGCTGGATGATCGTCGG	AGAAAGCCGGTGGGCAATAA	1547
*pmxD *	ATGAAAAAGGGCGGATGGCT	CTAGCCGTACAGCCGGGCGT	1734
*pmxE *	GCAAAATCCAACGCAGTGGT	ACGTTGAGAATGCGGTGTCT	1135
*spo0A *	ATGAAAATATACGCGATTGA	TTACGCCTGTGTTGCCACTT	699
*abrB *	TCAGTTTGTCACAGGTGTTG	ATGATGAAATCCACTGGATA	255
*ectB *	ATGAACACATTCGAAACACT	TACGCCCAGCACGTTCAATA	1145
*sfp *	TTGCAAAAAATTGAGGTATT	TCAGGACACCTTATTCTCAA	804

**Table 2:** Sequences of primer pairs for real-time PCR analysis of gene expression.

Genes	Nucleotide sequences (5′-3′)		Product sizes (bp)
	Forward primers	Reverse primers	
*pmxA *	TCAACTCGCTCAGAAGCGTT	TTGTACGGAAACCGACGGAG	105
*pmxB *	ATGAAATCTTTGTTTGAAAA	CCAGGACGTACACCCTCAAC	111
*pmxC *	TATTCCCGAGCTCATCACGC	TCGGAAGCGAACGACCATTT	107
*pmxD *	TGTTCGTTCAACGCCTCGTA	GCTTGCAAACGCTCGGTAAA	118
*pmxE *	CACTTTGCCTGAAACGACCG	GCCAGAATGCGTTCATACCG	111
*spo0A *	TCGCAGAATCCCGCAACATA	CGGTTGTGGAGTCAGGTTCA	103
*abrB *	AAATACGGAACAGCCCGTCC	TCGCTCGCCTGTCTTCAAAT	114
*ectB *	CAGTGGATACGGTCTGCCAA	CTCCGACAAACGCTAGCTGA	113
*sfp *	GTACCTCCTGCGCAAAGTGA	CACGACAGAGGGCTTTACGA	110
